# Poor-Prognosis Metastatic Cancers in Adolescents and Young Adults: Incidence Patterns, Trends, and Disparities

**DOI:** 10.1093/jncics/pkab039

**Published:** 2021-04-27

**Authors:** Jessica Sheth Bhutada, Amie Hwang, Lihua Liu, Dennis Deapen, David R Freyer

**Affiliations:** 1 Cancer and Blood Disease Institute, Children’s Hospital Los Angeles, Los Angeles, CA, USA; 2 USC Norris Comprehensive Cancer Center, Los Angeles, CA, USA; 3 Los Angeles Cancer Surveillance Program, Los Angeles, CA, USA; 4 Department of Preventive Medicine, Keck School of Medicine, University of Southern California, Los Angeles, CA, USA; 5 Department of Pediatrics, Keck School of Medicine, University of Southern California, Los Angeles, CA, USA; 6 Department of Medicine, Keck School of Medicine, University of Southern California, Los Angeles, CA, USA

## Abstract

**Background:**

For adolescents and young adults (AYAs, aged 15-39 years) with cancer, metastatic disease at diagnosis is the strongest predictor of mortality, but its associations with age and sociodemographic factors are largely unexplored.

**Methods:**

Using Surveillance, Epidemiology, and End Results Program data from 2000 to 2016, we collected incident cases of poor-prognosis metastatic cancer (5-year survival < 50%) and compared the proportion, incidence, time trends, and incidence rate ratios for race and ethnicity, sex, and socioeconomic status among AYAs, middle-aged adults (aged 40-64 years) and older adults (aged 65-79 years).

**Results:**

From 2000 to 2016, a total of 17 210 incident cases of poor-prognosis metastatic cancer were diagnosed in AYAs, 121 274 in middle-aged adults, and 364 228 in older adults. Compared with older patients, the proportion of AYAs having metastatic disease was equivalent or substantially lower in nearly every site except stomach and breast cancers, which were statistically significantly higher for AYAs compared with middle-aged and older adults (stomach: 57.3% vs 46.4% and 39.5%; breast: 6.6% vs 4.4% and 5.6%, respectively; 2-sided *P* < .001 for all comparisons). Incidence rates rose significantly faster among AYAs for breast, stomach, and kidney cancers and among AYAs and middle-aged adults for colorectal cancer. Markedly higher incidence rate ratios were noted for AYA racial and ethnic minorities with breast, stomach, and especially kidney cancer, where only non-Hispanic Black AYAs were at considerably higher risk. For most sites, incidence rate ratios were higher among male patients and individuals of low socioeconomic status across age groups.

**Conclusions:**

For most cancers, AYAs are not more likely to present with metastases than middle-aged and older adults. Further investigation is warranted for the disproportionate rise in incidence of metastatic breast, stomach, and kidney cancer among AYAs and their excess burden among AYA racial and ethnic minorities. The rising incidence of colorectal cancer among AYAs and middle-aged adults remains an additional concern.

During the past 3 decades, studies of cancer in adolescents and young adults (AYAs, 15-39 years of age) have documented multiple age-related disparities including poorer survival improvement (1,2), unfavorable tumor biology (3), excess treatment-related toxicity (4), limited access to appropriate care (5), low participation in clinical trials (6), heightened financial vulnerability (7), and unique psychosocial needs (8). As a result, increased resources for enhancing AYA cancer care and research have led to improved outcomes (9-12), such that 5-year survival for all AYAs combined now exceeds 84% (13-18).

Unfortunately, substantially poorer survival continues to plague certain AYA subsets defined by diagnosis, histology, stage, sex, race and ethnicity, and socioeconomic status (SES) (13,14,16,19-21). Of all these, having metastatic disease at diagnosis portends the worst outcome. Among AYAs with most types of metastatic cancer, 5-year survival fails to reach 40% and is much lower for many. For metastatic breast and colorectal carcinoma, 5-year survival is only 15%-20% (16,19). For metastatic melanoma and metastatic carcinomas of the kidney, stomach, and lung, 5-year survival is less than 10% (16,19). Adjusted for other factors, AYAs diagnosed with metastatic cancer have a 6-fold greater mortality than those with localized disease (13). For breast, lung, stomach, and colorectal carcinoma, as well as soft tissue sarcoma, AYAs with metastatic disease have an 8- to 14-fold higher risk of death than localized tumors (13). For AYAs with metastatic melanoma and metastatic carcinomas of the uterus and kidney, mortality risk is more than 30-fold higher (13). Independent of cancer stage, AYAs who are racial and/or ethnic minorities, male, or low SES have statistically significantly poorer survival, a disparity that actually worsened from 1988 to 2014 for non-Hispanic Blacks (NHBs) and low SES (13,17). Collectively, these observations indicate that a deeper understanding of metastatic cancer is needed to achieve further meaningful improvements in AYA survival, especially in the context of high-risk sociodemographic subgroups.

Distinct patterns of sociodemographic and biological factors are associated with certain high-risk metastatic and locoregional cancers in AYAs. For example, younger Black women are more likely to develop the HER2-, estrogen-, and progesterone-negative (triple negative) form of metastatic breast cancer (22-24). Racial and ethnic minorities and younger adults with colorectal carcinoma are more likely to have aggressive histologic features and develop left-sided tumors (25-29). The incidence of metastatic breast, colorectal, and uterine carcinoma is rising in AYAs, although the role of race and ethnicity is not yet fully characterized (30). Further, AYAs with cancer, especially minorities and low SES, are disproportionately underinsured with impaired access to care possibly resulting in delayed diagnosis, advanced stage disease, and lower survival (31-33). Despite this, studies of cancer incidence in AYAs have not comprehensively evaluated patterns of metastatic disease and their potential associations with sociodemographic factors (30,34).

Given these gaps and the profound impact of metastatic disease on survival, we used recent data from the Surveillance, Epidemiology, and End Results (SEER) program to compare the incidence patterns of several metastatic cancers and their sociodemographic relationships between AYAs vs older patients. Our overall objective was to determine whether age-related differences exist within and across cancers by sociodemographic factors. Using this landscape approach, we postulated our results would yield insights about the AYA patient as a cancer host and identify AYA subgroups at greatest risk for presenting with metastatic disease, findings that could have potential implications for AYA cancer care, prevention, and research.

## Methods

### Data Source and Cancer Selection

This was a population-based study utilizing SEER-18 registry data. Patients were aged 15-79 years when diagnosed with selected poor-prognosis, metastatic primary malignancies between January 2000 and December 2016. Patients with subsequent primary cancers were excluded. Metastatic cancers were defined as poor prognosis by having 5-year survival less than 50%; this included bone tumors (osteosarcoma, chondrosarcoma, Ewing sarcoma, and others), melanoma, rhabdomyosarcoma, other soft tissue sarcomas, and carcinomas of the breast, cervix, uterus, ovary, colon-rectum, kidney, lung, and stomach. Rhabdomyosarcoma was evaluated separately as it is clinically and biologically distinct from other soft tissue sarcomas (35). Cervical and uterine cancers were examined separately because of differences in biology, risk factors, and screening. Kaposi sarcoma and non-Hodgkin lymphoma were excluded because of their distinct HIV-associated epidemiology (13,36).

### Variable Definitions

Patients were divided into 3 age groups: AYA (15-39 years), middle-aged adults, and older adults. Middle-aged adults were defined as aged 40-64 years for all cancers except for breast and colorectal cancer (both 40-49 years) and lung (40-54 years). Older adults were defined as aged 65-79 years except for breast and colorectal cancer (both 50-74 years) and lung (55-79 years); in these sites, upper and lower ages were aligned with current US Preventive Services Task Force screening recommendations (37). For each case, stage at diagnosis (localized, regional, distant, unknown), sex (male, female), age at diagnosis, race, and ethnicity (non-Hispanic White [NHW]; NHB; non-Hispanic Asian and Pacific Islander; and Hispanic [all races]) were assessed. Population denominator estimates were created by SEER using an iterative proportional fitting algorithm that allocates multiracial populations to 1 of 4 single race categories at the census tract level (38). Metastatic disease was denoted by “distant” stage disease, defined by the SEER coding rule as “tumor which has spread to body areas distant or remote from the primary tumor” (39). For all age groups, the primary cancer site was identified using the SEER AYA site recode, which was developed specifically for the AYA population to reflect the impact of histology over topography (40). Application of the histologically defined AYA site recode to middle-aged and older adults maximized relevance of cross-age comparisons through more accurate identification of the cancer types of interest.

The SEER census tract level SES index is a time-dependent composite score constructed from 7 relevant census tract variables (41), including median household income, median house value, median rent, percent less than 150% of poverty line, education index, percent working class, and percent unemployed (42,43). The SES indices are calculated for each year using census data and a series of American Community Survey 5-year estimates. SES scores are subsequently categorized into tertiles with equal populations across the entire SEER catchment area. Tertiles were chosen instead of quintiles to optimize case numbers for all cancers and were accessed through the SEER specialized census-tract level and rurality database.

### Statistical Analyses

Incidence data were obtained using SEER*Stat software version 8.3.6. Proportion of metastatic compared with localized and regional disease was calculated for each cancer site and compared between age groups. χ^2^ analysis with post hoc pairwise comparisons utilizing an adjusted 2-sided *P* value of .025 (Bonferroni adjustment) was performed to determine if the proportion of metastatic disease was statistically significantly different between AYAs and middle-aged adults and AYAs and older adults. Age-adjusted incidence rates of metastases were estimated using the 2000 US standard population at 5-year age intervals and are reported as counts per 100 000 population at risk (44). Within each age group and by cancer, incidence rate ratios were calculated by comparing incidence rates for sociodemographic subgroups of interest with a designated reference group. Reference groups for race and ethnicity, sex, and SES subgroups were defined as NHWs, female, and high SES, respectively. Incidence rate ratios for sociodemographic subgroups were then compared between age groups. Standard errors and 95% confidence intervals were calculated using the Tiwari et al. modification (45). To evaluate trends in incidence rates of metastatic disease from 2000 to 2016, the age-adjusted annual percent change was calculated using the weighted least squares method with 95% confidence intervals for cancers with sufficient cases, defined as more than 25 cases per year, in each age group (44,46). Statistical significance of trends was evaluated for the 2-sided null hypothesis that the annual percent change was equal to zero (*P* < .05).

## Results

### Relative Proportion and Incidence of Metastatic Cancers

**Figure 1.**
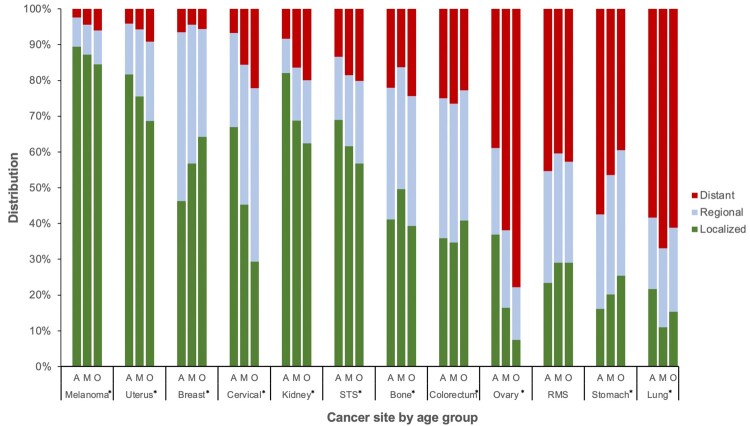
Stage distribution by cancer site and age group: Surveillance, Epidemiology, and End Results Program (2000-2016). Asterisk (*) indicates overall χ^2^ with 2-sided *P* < .001. A = adolescent and young adult; M = middle-aged adult; O = older adult; RMS = rhabdomyosarcoma; STS = soft tissue sarcoma (excluding rhabdomyosarcoma and Kaposi sarcoma).

From 2000 to 2016, a total of 17 210 incident cases of poor-prognosis metastatic cancer were diagnosed in AYAs, 121 274 in middle-aged adults, and 364 228 in older adults. The proportion of metastatic disease varied across cancer types, ranging from less than 10% for melanoma to more than 50% for ovarian, stomach, and lung cancer (Figure 1). For nearly all cancers, the proportion of AYAs with metastatic disease was statistically significantly lower than both middle-aged and older adults, except for breast and stomach cancer where it was higher for AYAs (Supplementary Table 1, available online). Within ovarian cancer, only 38.9% of AYAs had metastatic disease compared with 77.3% of older adults (*P* < .001). In contrast, within stomach cancer, 57.3% of AYAs presented with metastatic disease compared with 46.4% and 39.5% of middle-aged and older adults, respectively (*P* < .001). Within colorectal cancer, the proportion of metastatic disease was slightly lower in AYAs than middle-aged adults (aged 40-49 years) but, for both groups, was higher than older adults.

In general, age-adjusted incidence rates for metastatic cancers were lower for AYAs than for middle-aged and older adults, particularly among breast, colorectal, ovarian, and lung (Table 1). Among AYAs, breast cancer had the highest incidence rate of metastatic disease at 1.13 cases per 100 000, more than 1.5 to 2 times that of metastatic colorectal, ovarian, and lung cancer. Rhabdomyosarcoma demonstrated the lowest incidence rate in all age groups.

**Table 1.** Age-adjusted incidence rate per 100 000 of metastatic disease by cancer site and age group^a^

Cancer site	AYA		Middle-aged adults		Older adults	
	Rate (95% CI)	No.	Rate (95% CI)	No.	Rate (95% CI)	No.
Breast						
Distant	1.13 (1.09 to 1.18)	2672	5.29 (5.16 to 5.43)	5600	12.50 (12.33 to 12.67)	22 525
All stages	17.38 (17.21 to 17.55)	40 606	120.77 (120.12 to 121.43)	127 592	221.98 (221.29 to 222.67)	399 042
Colorectal						
Distant	0.82 (0.79 to 0.85)	3917	5.29 (5.20 to 5.39)	11 152	16.88 (16.74 to 17.02)	57 369
All stages	3.27 (3.21 to 3.32)	15 657	20 (19.79 to 20.18)	42 089	74.57 (74.23 to 74.87)	252 408
Ovarian						
Distant	0.59 (0.56 to 0.62)	1409	7.93 (7.81 to 8.04)	19 811	22.11 (21.76 to 22.47)	15 295
All stages	1.51 (1.46 to 1.56)	3623	12.95 (12.81 to 13.09)	32 011	28.54 (28.13 to 28.93)	19 780
Lung						
Distant	0.55 (0.54 to 0.59)	2645	13.44 (13.31 to 13.57)	43 035	90.4 (90.04 to 90.77)	239 957
All stages	0.96 (0.93 to 0.98)	4530	20.08 (19.9 to 20.24)	64 339	148.62 (148.15 to 149.10)	392 371
Cervix						
Distant	0.40 (0.37 to 0.42)	941	1.68 (1.63 to 1.73)	4026	1.86 (1.76 to 1.97)	1304
All stages	5.78 (5.68 to 5.88)	13 895	11.22 (11.10 to 11.37)	25 830	8.38 (8.16 to 8.59)	5876
Stomach						
Distant	0.34 (0.33 to 0.36)	1647	2.59 (2.54 to 2.63)	12 591	8.1 (7.93 to 8.24)	10 128
All stages	0.6 (0.58 to 0.62)	2875	5.55 (5.48 to 5.61)	27 117	20.55 (20.30 to 20.81)	25 668
Soft tissue sarcoma						
Distant	0.24 (0.23 to 0.25)	1194	0.83 (0.81 to 0.86)	3997	1.72 (1.65 to 1.79)	2175
All stages	1.79 (1.76 to 1.83)	8920	4.57 (4.51 to 4.63)	21 561	8.56 (8.4 to 8.72)	10 791
Bone						
Distant	0.18 (0.17 to 0.19)	919	0.12 (0.11 to 0.13)	542	0.24 (0.21 to 0.27)	301
All stages	0.82 (0.79 to 0.84)	4188	0.71 (0.69 to 0.74)	3312	0.97 (0.92 to 1.03)	1234
Melanoma						
Distant	0.15 (0.13 to 0.15)	720	0.89 (0.87 to 0.92)	4357	2.29 (2.2 to 2.37)	2869
All stages	6 (5.93 to 6.07)	29 604	20.63 (20.49 to 20.75)	97 770	37.82 (37.48 to 38.16)	47 907
Kidney						
Distant	0.13 (0.12 to 0.14)	602	2.35 (2.3 to 2.39)	11 616	6.74 (6.60 to 6.89)	8530
All stages	1.51 (1.47 to 1.54)	7165	14.54 (14.43 to 14.65)	70 523	33.6 (33.29 to 33.93)	42 777
Uterus						
Distant	0.09 (0.08 to 0.1)	216	1.71 (1.66 to 1.76)	4393	5.41 (5.24 to 5.59)	3777
All stages	2.24 (2.17 to 2.29)	5266	30.08 (29.86 to 30.29)	76 491	58.74 (58.18 to 59.32)	41 324
Rhabdomyosarcoma						
Distant	0.06 (0.05 to 0.06)	311	0.04 (0.03 to 0.04)	174	0.08 (0.06 to 0.10)	101
All stages	0.13 (0.12 to 0.14)	692	0.09 (0.08 to 0.1)	431	0.18 (0.16 to 0.21)	235

a Surveillance, Epidemiology, and End Results Program (2000-2016). AYA = adolescents and young adults; CI = confidence interval.

### Time Trends

**Figure 2.**
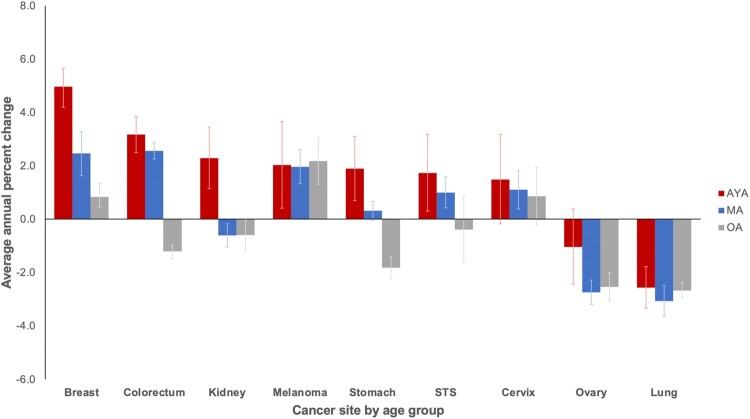
Average annual percent change of metastatic disease incidence rates by cancer site: Surveillance, Epidemiology, and End Results Program (2000-2016). Bone, rhabdomyosarcoma, and uterus not displayed because of insufficient cases (see the Methods section). Error bars indicate 95% confidence intervals. AYA = adolescent and young adult; MA = middle-aged adult; OA = older adult; STS = soft tissue sarcoma (excluding rhabdomyosarcoma and Kaposi sarcoma).

Trends in annual percent change from 2000 to 2016 indicate incidence rates of metastatic disease are rising faster in AYAs than middle-aged and older adults for all cancer sites except melanoma, lung, and ovarian and statistically significantly more so for breast, stomach, and kidney cancer (Figure 2; Supplementary Table 2, available online). For metastatic breast cancer, the magnitude of the increase was statistically significantly larger among AYAs at almost 5% per year compared with increases of 2% and less than 1% among middle-aged and older adults, respectively. For metastatic colorectal cancer and soft tissue sarcomas, incidence rates increased statistically significantly for both AYAs and middle-aged adults and, for stomach cancer, increased statistically significantly only among AYAs but declined among older adults. Specifically, within AYAs with colorectal cancer, the incidence of metastatic rectal cancer rose annually at a faster rate than colon cancer (4.5% vs 2.6%, respectively). This is in contrast to middle-aged adults, where the annual percent change was almost identical for both colon and rectal cancer (2.4% and 2.8%, respectively) and older adults where the rates decreased by 1.7% and 0.33%, respectively. Within kidney cancer, AYAs were the only group where the incidence of metastatic disease increased.

### Sociodemographic Patterns

**Figure 3.**
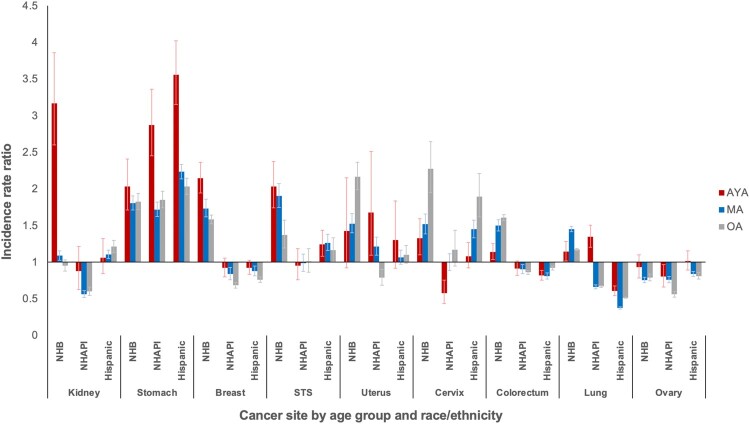
Incidence rate ratios of metastatic disease by race and ethnicity and age group: Surveillance, Epidemiology, and End Results Program (2000-2016). Reference group is non-Hispanic White. Cancers with subgroup population denominators less than 50 000 not displayed to protect confidentiality. Error bars indicate 95% confidence intervals. AYA = adolescent and young adult; MA = middle-aged adult; NHAPI = non-Hispanic Asian and Pacific Islander; NHB = non-Hispanic Black; OA = older adult; STS = soft tissue sarcoma (excluding rhabdomyosarcoma and Kaposi sarcoma).

To investigate potential risk differences by sociodemographic factors, incidence rate ratios were calculated among age groups within each cancer. Racial and ethnic differences in the incidence of metastatic cancer varied substantially between cancer type and age (Figure 3). The most striking differences were clustered in stomach cancer, where all racial and ethnic minorities in all age groups were at statistically significantly higher risk than NHWs. However, within every racial and ethnic subset, the risk for metastatic stomach cancer was highest for AYAs with approximately 2- to 3.5-fold greater risk. Across age groups and in multiple cancers, NHBs were generally at higher risk than NHWs and other minority groups. However, among NHBs, this risk was highest for AYAs with breast and, particularly, kidney cancer, where the risk for NHB AYAs was more than 3-fold higher than any other age or racial and ethnic group. In soft tissue sarcoma, NHB AYAs and middle-aged adults were similarly affected though to a lesser extent. Increased risks were seen for racial and ethnic minorities with uterine, cervical, and colorectal cancer, but the risk was generally lower for AYAs compared with middle-aged and older adults.

**Figure 4.**
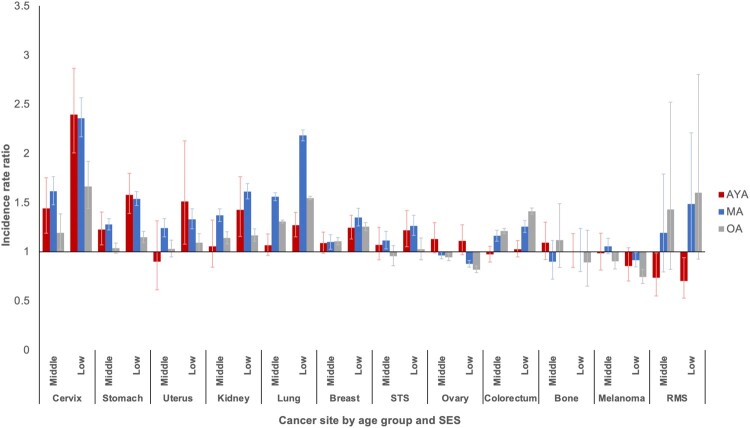
Incidence rate ratios of metastatic disease by socioeconomic status (SES) and age group: Surveillance, Epidemiology, and End Results Program (2000-2016). Reference group is highest SES tertile. Error bars indicate 95% confidence intervals. AYA = adolescent and young adult; MA = middle-aged adult; OA = older adult; RMS = rhabdomyosarcoma; STS = soft tissue sarcoma (excluding rhabdomyosarcoma and Kaposi sarcoma).

Overall, there was a consistent pattern of excess risk for developing metastatic cancer among patients of middle and low SES compared with those of high SES (Figure 4). The excess risk for middle and low SES was highest for metastatic cancer of the cervix, stomach, uterus, kidney, and lung. Within the middle and low SES groups, the risk for AYAs was generally equal to or lower than their older counterparts.

**Figure 5.**
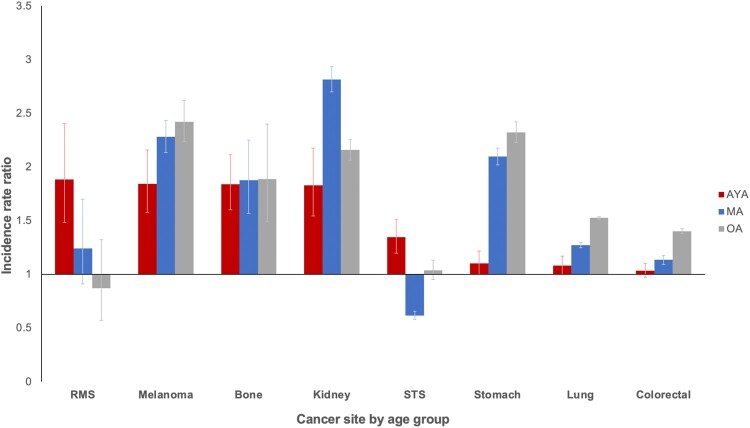
Incidence rate ratios of metastatic disease for male patients by age group: Surveillance, Epidemiology, and End Results Program (2000-2016). Reference group is female. Error bars indicate 95% confidence intervals. AYA = adolescent and young adult; MA = middle-aged adult; OA = older adult; RMS = rhabdomyosarcoma; STS = soft tissue sarcoma (excluding rhabdomyosarcoma and Kaposi sarcoma).

Finally, there was an approximately 2-fold greater risk for male patients in almost every type of metastatic cancer across age groups (Figure 5). For metastatic rhabdomyosarcoma and soft tissue sarcoma, the excess risk for male AYAs was notably higher than older male adults. For other cancers, the increased risk for male patients was relatively lower for AYAs compared with middle-aged and older adults.

## Discussion

The presence of metastases at diagnosis is the strongest and most consistent predictor of mortality for AYAs with cancer (13). In this study, we used an age-stratified, population-based approach to characterize the incidence patterns, time trends, and disparities of high-risk metastatic cancer for AYAs and middle-aged and older adults by sociodemographic factors known to be associated with lower survival. Three major conclusions can be drawn from these results. First, for nearly all cancers, the proportion of AYAs that present with metastatic disease is similar to or statistically significantly lower than middle-aged and older adults, with the prominent exceptions of breast and, especially, stomach cancer. Second, for nearly all cancers, the incidence of metastatic disease is rising over time in AYAs and middle-aged and older adults but considerably faster among AYAs for breast, stomach, and kidney cancer and among AYAs and middle-aged adults for colorectal cancer. Third, in most age groups and most cancers, the risk for having metastatic disease is greatest among those who are NHB, Hispanic, low SES, and/or male. Taken together, these findings suggest that, with the important exceptions of breast, colorectal, kidney, and stomach cancer discussed below, the burden of metastatic cancer in AYAs is largely similar to that in older patients.

Our finding of excess metastatic breast cancer in NHB AYAs aligns with recent literature describing higher rates of triple-negative breast cancer, a known aggressive molecular subtype associated with increased metastases, in NHB women of all ages (47-50). The concurrent 20% higher risk for metastases we observed among AYAs with lower SES may implicate a connection between low SES and NHB race, as well as potential environmental and biological associations that promote development of aggressive disease. Studies that have examined the association between neighborhood disadvantage and the presence of aggressive features such as triple-negative breast cancer suggest potential biological links mediated through stress reactivity and stress-induced inflammation (51). However, another study found that although low SES contributes to racial disparities in stage at diagnosis and survival among triple-negative breast cancer, the incidence of triple-negative breast cancer did not vary by SES (52). These relationships require further in-depth study and novel approaches to investigate the interplay between race and ethnicity, tumor biology, and the built environment associated with SES.

Our results regarding metastatic colorectal cancer are consistent with other studies showing the incidence is rising disproportionately among AYAs and younger middle-aged adults (53). Whereas studies exploring sociodemographic characteristics of colorectal cancer in AYAs have identified an increasing incidence of nonmetastatic disease in young, NHB, low SES individuals (25,27), our results show the incidence rate is statistically significantly higher among NHBs for all age groups, including AYAs, but similar for sex and SES level. This suggests the increasing incidence rate of metastatic disease in AYAs and younger middle-aged adults may be related to age-specific host or environmental factors that have changed over time and predispose this population, particularly NHBs, to early onset colorectal cancer. Notably, within AYAs, the disproportionate rise in metastatic rectal cancer compared with colon cancer highlights the need for further studies to better understand the etiology and impact of this trend.

In stomach cancer, we found a disproportionately rising incidence and overrepresentation of metastatic disease among AYAs. In addition to the possibilities of biologically aggressive disease or environmental factors, potential explanations in this age group include delayed diagnosis because of clinically subtle presentation, neglect of early symptoms, or barriers to accessing care. Additional research is needed to delineate these factors. Our subgroup analysis showed that AYA minorities, especially Hispanics and non-Hispanic Asian and Pacific Islanders, are disproportionately burdened with metastatic disease. These findings are consistent with recent reports documenting excess stomach cancer among Hispanics (54). For stomach cancer, potential mechanisms of metastases have been tied to tumor microenvironment as well as dietary factors, *Hpylori* infection, and EBV infection (55). Studies showing increased risk of *Hpylori* infections in Hispanic adults may explain the disproportionate burden of metastases seen in Hispanic patients (56). Given that Hispanics with stomach cancer have poorer outcomes than NHWs, this vulnerable group of AYAs may be candidates for targeted screening, preventive, and therapeutic strategies as expert groups develop consensus guidelines (56,57). A higher level of clinical suspicion among primary care physicians caring for high-risk AYAs with persistent symptoms consistent with stomach cancer may also be warranted.

In this study, kidney cancer exhibited the most striking age-related disparity. We discovered a recent, marked rise in incidence rate of metastatic disease found only in AYAs and almost exclusively among NHBs. We determined this observation was not explained by interregistry variation or SEER coding changes redefining metastatic renal carcinoma during our study period (58). Whereas a rising incidence of all-stage (34) and nonmetastatic renal carcinoma has been described and attributed to overdiagnosis through incidental radiographic findings (59,60), this does not explain our results with metastatic disease, which is more likely to be symptomatic and clinically detected. The known associations between renal cell carcinoma, smoking (61), obesity, and hypertension (62), in conjunction with data indicating these risk factors occur disproportionately in NHBs (63-65), may explain our observation through potential differences in host biology and health behaviors. Additionally, the higher incidence of translocation-positive renal cell carcinoma, an aggressive subtype associated with metastatic disease, in AYAs may contribute to the rising incidence of metastatic disease (66,67).

This study has notable strengths and some limitations. A key strength is the use of SEER registry data, a robust and reliable resource that permits identification of broad trends across a variety of cancers, including rare tumors such as rhabdomyosarcoma, a biologically distinct form of sarcoma prone to present with metastases in the AYA age group. Although evaluation of sociodemographic trends was limited in rhabdomyosarcoma because of the small number of patients in this national sample, this study highlights the need for large-scale collaborations to better understand risk factors in these rare tumors. Another strength is including middle- and older-aged adults as separate comparison groups, which provides a more nuanced view of similarities and differences across the age spectrum of adulthood. Potential limitations are those inherent to registry-based research, including possible misclassification of race or ethnicity provided by the reporting site and use of area-based SES rather than individual level. Additionally, the SEER registry currently does not delineate cancer sites by molecular subtype, and detailed patient-level treatment and clinical data are not available. These considerations limit in-depth study of biologically focused characteristics (68). For example, rhabdomyosarcoma was included in our analysis as an important AYA cancer, yet comparison with older patients is challenging to interpret in light of subtype differences that vary by age, such as *PAX3-FOXO1* fusions among AYAs and pleomorphic histology among middle-aged and older adults (69). In this and other forms of metastatic cancer where differences in clinical behavior exist between AYAs and middle-aged and older adults, future studies should make every effort to account for histological and molecular subtypes for more meaningful comparisons.

Nonetheless, provisional inferences can be drawn. The fact that incidence patterns of metastatic cancer in AYAs are largely similar to older patients suggests that age is not the decisive factor for developing advanced stage disease in most malignancies. Concerns have been raised about AYAs being generally more prone to developing poor-prognosis cancers (3) and/or having delayed diagnosis resulting in late-stage disease (70). Given that we did not observe a consistent pattern of excess metastatic disease in AYAs across many cancers and sociodemographic groups, our results support the need for a more nuanced and cancer-specific approach. In particular, AYAs with metastatic breast, colorectal, stomach, and kidney cancer should be prioritized for more in-depth investigation of potential risk factors that explain the rising incidence, increased metastatic burden, and/or specific subgroup disparities observed in this study. Breast and colorectal cancer are particularly relevant, as these 2 cancers alone account for almost one-fourth of all cancer-related deaths in AYAs (71). Ultimately, such research could suggest a role for enhanced cancer detection or prevention strategies, and/or when coupled with similar survival analyses, indicate the relative importance of host, tumor, treatment, and/or health behaviors as outcome determinants.

## Funding

This work was supported by the John H. Richardson Endowed Fellowship Award through the Achievement Rewards for College Scientists Foundation Los Angeles Founder Chapter (JSB). The collection of cancer incidence data used in this study was supported by the California Department of Public Health pursuant to California Health and Safety Code Section 103885; the Centers for Disease Control and Prevention’s National Program of Cancer Registries, under cooperative agreement 5NU58DP003862-04/DP003862; the National Cancer Institute’s SEER Program under contract HHSN261201000140C awarded to the Cancer Prevention Institute of California, contract HHSN261201000035C awarded to the University of Southern California, and contract HHSN261201000034C awarded to the Public Health Institute.

## Notes

**Role of funder:** The study sponsor had no role in the study design in the collection, analysis, and interpretation of data, the writing of the manuscript, and the decision to submit the manuscript for publication.

**Disclosures:** The authors have no disclosures or conflicts of interest to report.

**Author contributions:** Conceptualization: all authors; Data curation: JSB; Formal analysis: JSB; Supervision: DRF; Visualization: JSB. DRF; Writing—Original Draft: JSB, DRF; Writing—Review & Editing: all authors.

**Acknowledgements:** This study would not have been possible without the hard work and dedication of cancer registrars and staff across all SEER registries.

**Disclaimers:** The ideas and opinions expressed herein are those of the author(s) and do not necessarily reflect the opinions of the State of California, Department of Public Health, the National Cancer Institute, and the Centers for Disease Control and Prevention or their contractors and subcontractors.

## Data Availability

Data was accessed from the SEER Census-Tract Level SES and Rurality Database and is publicly available upon request at https://seer.cancer.gov/seertrack/data/request/.

## Supplementary Material

pkab039_Supplementary_Data

## References

[1] Adolescent and Young Adult Progress Review Group. Closing the Gap: Research and Care Imperatives for Adolescents and Young Adults with Cancer. Bethesdam, MD: US Department of Health and Human Services, National Institutes of Health, National Cancer Institute, LIVESTRONG Young Adult Alliance; 2006.

[2] Barr RD , Ferrari A, Ries L, et al Cancer in adolescents and young adults. JAMA Pediatr. 2016;170(5):495–501. 26999630 10.1001/jamapediatrics.2015.4689

[3] Tricoli J , Bleyer A. Adolescent and young adult cancer biology. Cancer J. 2018;24(6):267–274. 30480571 10.1097/PPO.0000000000000343

[4] Bukowinski AJ , Burns KC, Parsons K, et al Toxicity of cancer therapy in adolescents and young adults (AYAs). Semin Oncol Nurs. 2015;31(3):216–226. 26210200 10.1016/j.soncn.2015.05.003

[5] Albritton KH , Wiggins CH, Nelson HE, et al Site of oncologic specialty care for older adolescents in Utah. J Clin Oncol. 2007;25(29):4616–4621. 17925557 10.1200/JCO.2006.08.4103

[6] Freyer DR , Seibel NL. The clinical trials gap for adolescents and young adults with cancer: recent progress and conceptual framework for continued research. Curr Pediatr Rep. 2015;3(2):137–145. 30613438 10.1007/s40124-015-0075-y PMC6319956

[7] Salsman JM , Bingen K, Barr RD, et al Understanding, measuring, and addressing the financial impact of cancer on adolescents and young adults. Pediatr Blood Cancer. 2019;66(7):e27660. 30756484 10.1002/pbc.27660 PMC6777708

[8] Zebrack B , Isaacson S. Psychosocial care of adolescent and young adult patients with cancer and survivors. J Clin Oncol. 2012;30(11):1221–1226. 22412147 10.1200/JCO.2011.39.5467

[9] Freyer DR , Felgenhauer J, Perentesis J; for the COG Adolescent and Young Adult Oncology Discipline Committee. Children’s Oncology Group’s 2013 blueprint for research: adolescent and young adult oncology. Pediatr Blood Cancer. 2013;60(6):1055–1058. 23424167 10.1002/pbc.24431 PMC4604044

[10] Orellana-Noia VM , Douvas MG. Recent developments in adolescent and young adult (AYA) acute lymphoblastic leukemia. Curr Hematol Malig Rep. 2018;13(2):100–108. 29442287 10.1007/s11899-018-0442-1

[11] Shaw PH , Reed DR, Yeager N, et al Adolescent and young adult (AYA) oncology in the United States. J Pediatr Hematol Oncol. 2015;37(3):161–169. 25757020 10.1097/MPH.0000000000000318

[12] Smith AW , Seibel NL, Lewis DR, et al. Next steps for adolescent and young adult oncology workshop: An update on progress and recommendations for the future. Cancer. 2016;122(7):988-999. 26849003 10.1002/cncr.29870 PMC7521143

[13] Moke DJ , Tsai K, Hamilton AS, et al Emerging cancer survival trends, disparities, and priorities in adolescents and young adults: a California cancer registry-based study. JNCI Cancer Spectr. 2019;3(2):pkz031. 31276099 10.1093/jncics/pkz031 PMC6597054

[14] Liu L , Moke DJ, Tsai K-Y, et al A reappraisal of sex-specific cancer survival trends among adolescents and young adults in the United States. J Natl Cancer Inst. 2019;111(5):509–518. 30321398 10.1093/jnci/djy140 PMC6510224

[15] Lewis DR , Seibel NL, Smith AW, et al Adolescent and young adult cancer survival. J Natl Cancer Inst Monogr. 2014;2014(49):228–235. 25417236 10.1093/jncimonographs/lgu019 PMC4841167

[16] Keegan THM , Ries LAG, Barr RD, et al; for the National Cancer Institute Next Steps for Adolescent and Young Adult Oncology Epidemiology Working Group. Comparison of cancer survival trends in the United States of adolescents and young adults with those in children and older adults. Cancer. 2016;122(7):1009–1016. 26848927 10.1002/cncr.29869

[17] Miller KD , Fidler‐Benaoudia M, Keegan TH, et al Cancer statistics for adolescents and young adults, 2020. CA Cancer J Clin. 2020;70(6):443–459. 32940362 10.3322/caac.21637

[18] Howlader N NA , Krapcho M, Miller D, eds. SEER Cancer Statistics Review. Bethesda, MD: National Cancer Institute; 1975-2017. https://seer.cancer.gov/csr/1975_2017/, based on November 2019 SEER data submission, posted to the SEER web site, April 2020. Accessed November 20, 2020.

[19] Liu L HA , Moke D, Tsai KY, eds. Cancer in Los Angeles County: Survival among Adolescents and Young Adults 1988-2014. Los Angeles Cancer Surveillance Program. University of Southern California; 2017. https://keck.usc.edu/cancer-surveillance-program/wp-content/uploads/sites/166/2016/03/aya_survival_2017.pdf

[20] Albano JD , Ward E, Jemal A, et al Cancer mortality in the United States by education level and race. J Natl Cancer Inst. 2007;99(18):1384–1394. 17848670 10.1093/jnci/djm127

[21] Kish JK , Yu M, Percy-Laurry A, et al Racial and ethnic disparities in cancer survival by neighborhood socioeconomic status in Surveillance, Epidemiology, and End Results (SEER) registries. J Natl Cancer Inst Monogr. 2014;2014(49):236–243. 25417237 10.1093/jncimonographs/lgu020 PMC4841168

[22] Tao L , Gomez SL, Keegan THM, et al Breast cancer mortality in African-American and non-Hispanic White women by molecular subtype and stage at diagnosis: a population-based study. Cancer Epidemiol Biomarkers Prev. 2015;24(7):1039–1045. 25969506 10.1158/1055-9965.EPI-15-0243 PMC4490947

[23] Sineshaw HM , Gaudet M, Ward EM, et al Association of race/ethnicity, socioeconomic status, and breast cancer subtypes in the National Cancer Data Base (2010-2011). Breast Cancer Res Treat. 2014;145(3):753–763. 24794028 10.1007/s10549-014-2976-9

[24] Keegan TH , Derouen MC, Press DJ, et al Occurrence of breast cancer subtypes in adolescent and young adult women. Breast Cancer Res. 2012;14(2):R55. 22452927 10.1186/bcr3156 PMC3446389

[25] Murphy CC , Wallace K, Sandler RS, et al Racial disparities in incidence of young-onset colorectal cancer and patient survival. Gastroenterology. 2019;156(4):958–965. 30521807 10.1053/j.gastro.2018.11.060 PMC6409160

[26] Stewart SL , Wike JM, Kato I, et al A population-based study of colorectal cancer histology in the United States, 1998-2001. Cancer. 2006;107(5 suppl):1128–1141. 16802325 10.1002/cncr.22010

[27] Holowatyj AN , Lewis MA, Pannier ST, et al Clinicopathologic and racial/ethnic differences of colorectal cancer among adolescents and young adults. Clin Transl Gastroenterol. 2019;10(7):e00059. 31259751 10.14309/ctg.0000000000000059 PMC6708666

[28] Wang R , Wang MJ, Ping J. Clinicopathological features and survival outcomes of colorectal cancer in young versus elderly: a population-based cohort study of SEER 9 Registries Data (1988-2011). Medicine (Baltimore). 2015;94(35):e1402. 26334895 10.1097/MD.0000000000001402 PMC4616510

[29] O’Connell JB , Maggard MA, Livingston EH, et al Colorectal cancer in the young. Am J Surg. 2004;187(3):343–348. 15006562 10.1016/j.amjsurg.2003.12.020

[30] Kehm RD , Yang W, Tehranifar P, et al 40 years of change in age- and stage-specific cancer incidence rates in US women and men. JNCI Cancer Spectr. 2019;3(3):pkz038. 31414075 10.1093/jncics/pkz038 PMC6686848

[31] Martin S , Ulrich C, Munsell M, et al Delays in cancer diagnosis in underinsured young adults and older adolescents. Oncologist. 2007;12(7):816–824. 17673613 10.1634/theoncologist.12-7-816

[32] Penumarthy NL , Goldsby RE, Shiboski SC, et al Insurance impacts survival for children, adolescents, and young adults with bone and soft tissue sarcomas. Cancer Med. 2020;9(3):951–958. 31838786 10.1002/cam4.2739 PMC6997066

[33] Keegan THM , Parsons HM, Chen Y, et al Impact of health insurance on stage at cancer diagnosis among adolescents and young adults. J Natl Cancer Inst. 2019;111(11):1152–1160. 30937440 10.1093/jnci/djz039 PMC6855930

[34] Scott AR , Stoltzfus KC, Tchelebi LT, et al Trends in cancer incidence in US adolescents and young adults, 1973-2015. JAMA Netw Open. 2020;3(12):e2027738. 33258907 10.1001/jamanetworkopen.2020.27738 PMC7709088

[35] Skapek SX , Ferrari A, Gupta AA, et al Rhabdomyosarcoma. Nat Rev Dis Primers. 2019;5(1):1. 30617281 10.1038/s41572-018-0051-2 PMC7456566

[36] Shiels MS , Cole SR, Wegner S, et al Effect of HAART on incident cancer and noncancer AIDS events among male HIV Seroconverters. J Acquir Immune Defic Syndr. 2008;48(4):485–490. 18614916 10.1097/QAI.0b013e31817dc42b PMC2805176

[37] US Preventive Services Task Force. Screening guidelines. https://www.uspreventiveservicestaskforce.org/uspstf/recommendation-topics/uspstf-and-b-recommendations. Accessed April 29, 2020.

[38] National Cancer Institute Surveillance, Epidemiology, and End Results Program SEER 18 Regs Nov 2018 Sub (2000-2016) National Cancer Institute, DCCPS, *Surveillance* Research Program, Surveillance Systems Branch. https://seer.cancer.gov/seerstat/databases/census-tract/index.html. Published 2020. Accessed April 29, 2020.

[39] National Cancer Institute Surveillance, Epidemiology, and End Results. Summary staging definition. https://training.seer.cancer.gov/staging/systems/summary/distant.html#:∼:text=Definition%3A,%2C%20disseminated%2C%20diffuse%2C%20metastatic. Accessed July 1, 2020.

[40] Barr RD , Holowaty EJ, Birch JM. Classification schemes for tumors diagnosed in adolescents and young adults. Cancer. 2006;106(7):1425–1430. 16544312 10.1002/cncr.21773

[41] Yu M , Tatalovich Z, Gibson JT, et al Using a composite index of socioeconomic status to investigate health disparities while protecting the confidentiality of cancer registry data. Cancer Causes Control. 2014;25(1):81–92. 24178398 10.1007/s10552-013-0310-1

[42] Liu L , Deapen D, Bernstein L. Socioeconomic status and cancers of the female breast and reproductive organs: a comparison across racial/ethnic populations in Los Angeles County, California (United States). Cancer Causes Control. 1998;9(4):369–380. 9794168 10.1023/a:1008811432436

[43] Yost K , Perkins C, Cohen R, et al Socioeconomic status and breast cancer incidence in California for different race/ethnic groups. Cancer Causes Control. 2001;12(8):703–711. 11562110 10.1023/a:1011240019516

[44] National Cancer Institute Surveillance, Epidemiology, and End Results. Trend algorithms. https://seer.cancer.gov/seerstat/WebHelp/seerstat.htm#Trend_Algorithms.htm. Accessed July 1, 2020.

[45] Tiwari RCC , Limin X, Zou Z. Efficient interval estimation for age-adjusted cancer rates. Stat Methods Med Res. 2006;15(6):547–569. 17260923 10.1177/0962280206070621

[46] Surveillance Research Program, National Cancer Institute SEER*Stat software version 8.3.6. seer.cancer.gov/seerstat

[47] Parise CA , Caggiano V. Disparities in race/ethnicity and socioeconomic status: risk of mortality of breast cancer patients in the California Cancer Registry, 2000-2010. *BMC Cancer*. 2013;13(1):449. 10.1186/1471-2407-13-449 PMC3850736 24083624

[48] Parise CA , Caggiano V. Differences in clinicopathologic characteristics and risk of mortality between the triple positive and ER+/PR+/HER2− breast cancer subtypes. Cancer Causes Control. 2019;30(5):417–424. 30879205 10.1007/s10552-019-01152-8

[49] Parise C , Caggiano V. Disparities in the risk of the ER/PR/HER2 breast cancer subtypes among Asian Americans in California. Cancer Epidemiol. 2014;38(5):556–562. 25172158 10.1016/j.canep.2014.08.001

[50] Bauer KR , Brown M, Cress RD, et al Descriptive analysis of estrogen receptor (ER)-negative, progesterone receptor (PR)-negative, and HER2-negative invasive breast cancer, the so-called triple-negative phenotype. Cancer. 2007;109(9):1721–1728. 17387718 10.1002/cncr.22618

[51] Saini G , Ogden A, McCullough LE, et al Disadvantaged neighborhoods and racial disparity in breast cancer outcomes: the biological link. Cancer Causes Control. 2019;30(7):677–686. 31111277 10.1007/s10552-019-01180-4 PMC7043809

[52] Hossain F , Danos D, Prakash O, et al Neighborhood social determinants of triple negative breast cancer. Front Public Health. 2019;7:18., 30834239 10.3389/fpubh.2019.00018 PMC6387917

[53] Siegel RL , Fedewa SA, Anderson WF, et al Colorectal cancer incidence patterns in the United States, 1974-2013. J Natl Cancer Inst. 2017;109(8):djw322. 10.1093/jnci/djw322 PMC6059239 28376186

[54] Islami F , Desantis CE, Jemal A. Incidence trends of esophageal and gastric cancer subtypes by race, ethnicity, and age in the United States, 1997-2014. Clin Gastroenterol Hepatol. 2019;17(3):429–439. 29902641 10.1016/j.cgh.2018.05.044

[55] Rawla P , Barsouk A. Epidemiology of gastric cancer: global trends, risk factors and prevention. Prz Gastroenterol. 2019;14(1):26–38. 30944675 10.5114/pg.2018.80001 PMC6444111

[56] Duma N. Gastric adenocarcinoma: clinicopathologic differences among Hispanics and non-Hispanic Whites. A single institution’s experience over 14 Years. Ann Gastroenterol. 2016;29(3):325–331.10.20524/aog.2016.0030. 27366033 10.20524/aog.2016.0030 PMC4923818

[57] Yang D , Hendifar A, Lenz C, et al Survival of metastatic gastric cancer: significance of age, sex and race/ethnicity. J Gastrointest Oncol. 2011;2(2):77–84. 22811834 10.3978/j.issn.2078-6891.2010.025 PMC3397601

[58] National Cancer Institute Surveillance, Epidemiology, and End Results. Historical staging and coding manuals. https://seer.cancer.gov/tools/codingmanuals/historical.html. Accessed November 20, 2020.

[59] Barr RD , Ries LAG, Lewis DR, et al; for the US National Cancer Institute Science of Adolescent and Young Adult Oncology Epidemiology Working Group. Incidence and incidence trends of the most frequent cancers in adolescent and young adult Americans, including “nonmalignant/noninvasive” tumors. Cancer. 2016;122(7):1000–1008. 26848808 10.1002/cncr.29867

[60] Znaor A , Lortet-Tieulent J, Laversanne M, et al International variations and trends in renal cell carcinoma incidence and mortality. Eur Urol. 2015;67(3):519–530. 25449206 10.1016/j.eururo.2014.10.002

[61] Hunt JD , Van Der Hel OL, McMillan GP, et al Renal cell carcinoma in relation to cigarette smoking: meta-analysis of 24 studies. Int J Cancer. 2005;114(1):101–108. 15523697 10.1002/ijc.20618

[62] Brennan P , Van Der Hel O, Moore LE, et al Tobacco smoking, body mass index, hypertension, and kidney cancer risk in central and Eastern Europe. Br J Cancer. 2008;99(11):1912–1915. 19034282 10.1038/sj.bjc.6604761 PMC2600689

[63] Lackland DT. Racial differences in hypertension: implications for high blood pressure management. Am J Med Sci. 2014;348(2):135–138. 24983758 10.1097/MAJ.0000000000000308 PMC4108512

[64] Liu X , Zhu T, Manojlovich M, et al Racial/ethnic disparity in the associations of smoking status with uncontrolled hypertension subtypes among hypertensive subjects. PLoS One. 2017;12(8):e0182807. 28793323 10.1371/journal.pone.0182807 PMC5549965

[65] Lincoln KD , Abdou CM, Lloyd D. Race and socioeconomic differences in obesity and depression among Black and non-Hispanic White Americans. J Health Care Poor Underserved. 2014;25(1):257–275. 24509025 10.1353/hpu.2014.0038 PMC4830390

[66] Ellis CL , Eble JN, Subhawong AP, et al Clinical heterogeneity of Xp11 translocation renal cell carcinoma: impact of fusion subtype, age, and stage. Mod Pathol. 2014;27(6):875–886. 24309327 10.1038/modpathol.2013.208

[67] Choo MS , Jeong CW, Song C, et al; for the Korean Renal Cancer Study Group. Clinicopathologic characteristics and prognosis of Xp11.2 translocation renal cell carcinoma: multicenter, propensity score matching analysis. Clin Genitourin Cancer. 2017;15(5):e819–e825. 28549862 10.1016/j.clgc.2017.04.015

[68] Pollock BH. What’s missing in the assessment of adolescent and young adult (AYA) cancer outcomes? J Natl Cancer Inst. 2020;112(10):975–976. 32123919 10.1093/jnci/djaa015 PMC7566536

[69] Leiner J , Le Loarer F. The current landscape of rhabdomyosarcomas: an update. Virchows Arch. 2020;476(1):97–108. 31696361 10.1007/s00428-019-02676-9

[70] Fardell JE , Patterson P, Wakefield CE, et al A narrative review of models of care for adolescents and young adults with cancer: barriers and recommendations. J Adolesc Young Adult Oncol. 2018;7(2):148–152. 29298105 10.1089/jayao.2017.0100

[71] National Cancer Institute Surveillance, Epidemiology, and End Results. Cancer stat facts: cancer among adolescents and young adults (AYAs) (ages 15-39). https://seer.cancer.gov/statfacts/html/aya.html#:∼:text=Based%20on%20estimates%20of%20new,for%205%20years%20after%20diagnosis. Accessed January 21, 2021.

