# Supplementary material for: Poor-Prognosis Metastatic Cancers in Adolescents and Young Adults: Incidence Patterns, Trends, and Disparities
Source: JNCI Cancer Spectr. 2021 Apr 27;5(3):pkab039. doi: 10.1093/jncics/pkab039 (PMC8266435; doi:10.1093/jncics/pkab039)
Supplement: pkab039_Supplementary_Data [file pkab039_supplementary_data.pdf]

SUPPLEMENTARY MATERIALS

Supplementary Table 1. Stage distribution by cancer type and age group[a]

Cancer Site	Stage	AYA	MA	OA	*P*		
		No. (%)	No. (%)	No. (%)	Overall Chi-Square	AYA vs MA	AYA vs OA
Melanoma	Distant	720 (2.4)	4357 (4.5)	2869 (6.0)	<0.0001	<0.0001	<0.0001
	Non-Distant	28884 (97.6)	93413 (95.5)	45038 (94.0)			
Uterus	Distant	216 (4.1)	4393 (5.7)	3777 (9.1)	<0.0001	<0.0001	<0.0001
	Non-Distant	5050 (95.9)	72098 (64.3)	37547 (90.9)			
Breast	Distant	2672 (6.6)	5600 (4.4)	22525 (5.6)	<0.0001	<0.0001[b]	<0.0001[b]
	Non-Distant	37934 (93.4)	121992 (95.6)	376517 (94.4)			
Cervix	Distant	941 (6.8)	4026 (15.6)	1304 (22.2)	<0.0001	<0.0001	<0.0001
	Non-Distant	12954 (93.2)	21804 (84.4)	4572 (77.8)			
Kidney	Distant	602 (8.4)	11616 (16.5)	8530 (19.9)	<0.0001	<0.0001	<0.0001
	Non-Distant	6563 (91.6)	58907 (83.5)	34247 (80.1)			
STS	Distant	1194 (13.4)	3997 (18.5)	2175 (20.2)	<0.0001	<0.0001	<0.0001
	Non-Distant	7726 (86.6)	17564 (81.5)	8616 (79.8)			
Bone	Distant	919 (21.9)	542 (16.4)	301 (24.4)	<0.0001	<0.0001[b]	0.0703
	Non-Distant	3269 (78.1)	2770 (83.6)	933 (75.6)			
Colorectal	Distant	3917 (25.0)	11152 (26.5)	57369 (22.7)	<0.0001	0.0003	<0.0001[b]
	Non-Distant	11740 (75.0)	30937 (73.5)	195039 (77.3)			
Ovarian	Distant	1409 (38.9)	19811 (61.9)	15295 (77.3)	<0.0001	<0.0001	<0.0001
	Non-Distant	2214 (61.1)	12200 (38.1)	4485 (22.7)			
RMS	Distant	311 (44.9)	174 (40.4)	101 (43.0)	0.3221	0.1326	0.6007
	Non-Distant	381 (55.1)	257 (59.6)	134 (57.0)			
Stomach	Distant	1647 (57.3)	12591 (46.4)	10128 (39.5)	<0.0001	<0.0001[b]	<0.0001[b]
	Non-Distant	1228 (42.7)	14526 (53.6)	15540 (60.5)			

Lung	Distant	2645 (58.4)	43035 (66.9)	239957 (61.2)	<0.0001	<0.0001	0.0001
	Non-Distant	1885 (41.6)	21304 (33.1)	152414 (38.8)			

[a] Surveillance, Epidemiology, and End Results Program (2000-2016). STS = Soft tissue sarcoma; RMS = Rhabdomyosarcoma; AYA = Adolescent and young adult; MA = Middle-aged adult; OA = Older adult.

[b] Indicates AYAs have a statistically significantly higher proportion of metastatic disease than comparator group.

Supplementary Table 2. Average annual percent change of metastatic disease incidence rates by cancer site[a]

Cancer Site	AYA		MA		OA	
	AAPC (95% CI)	P-Value	AAPC (95% CI)	P-Value	AAPC (95% CI)	P-Value
Breast	4.93 (4.19 to 5.66)	<0.001	2.46 (1.64 to 3.28)	<0.001	0.9 (0.46 to 1.35)	<0.001
Colorectum	3.17 (2.5 to 3.85)	<0.001	2.56 (2.26 to 2.86)	<0.001	-1.21 (-1.46 to -0.96)	<0.001
Kidney	2.29 (1.15 to 3.45)	<0.001	-0.61 (-1.04 to -0.18)	0.009	-0.59 (-1.14 to -0.03)	0.04
Melanoma	2.03 (0.42 to 3.66)	0.02	1.97 (1.35 to 2.6)	<0.001	2.19 (1.31 to 3.07)	<0.001
Stomach	1.89 (0.7 to 3.1)	0.004	0.32 (-0.03 to 0.67)	0.07	-1.81 (-2.23 to -1.39)	<0.001
STS	1.73 (0.3 to 3.18)	0.02	1 (0.43 to 1.58)	0.002	-0.38 (-1.61 to 0.86)	0.52
Cervix	1.49 (-0.17 to 3.18)	0.08	1.11 (0.39 to 1.83)	0.005	0.86 (-0.23 to 1.96)	0.11
Ovary	-1.03 (-2.43 to 0.38)	0.14	-2.74 (-3.19 to -2.28)	<0.001	-2.53 (-3.05 to -2.01)	<0.001
Lung	-2.56 (-3.34 to -1.78)	<0.001	-3.06 (-3.62 to -2.5)	<0.001	-2.67 (-2.95 to -2.38)	<0.001

[a] Surveillance, Epidemiology, and End Results Program (2000-2016). Bone, rhabdomyosarcoma and uterus not displayed due to insufficient cases (see Methods). Abbreviations: STS = Soft tissue sarcoma (excluding rhabdomyosarcoma and Kaposi sarcoma); AYA = Adolescent and young adult; MA = Middle-aged adult; OA = Older adult.
